
JOURNAL INFORMATION
==============================
NLM Title Abbreviation: Clin Infect Dis
Journal ID: Clin Infect Dis
NLM Journal ID: 9203213
Journal ID: cid

Clinical Infectious Diseases: An Official Publication of the Infectious Diseases Society of America

ISSN: 1058-4838
EISSN: 1537-6591

ARTICLE INFORMATION
==============================
PMCID: PMC7929054
PMID: 34492695
DOI: 10.1093/cid/ciab136
Article ID: ciab136
Article version: 1
Subjects: Correspondence, AcademicSubjects/MED00290

Evidence of SARS-CoV-2 reinfection without mutations in Spike protein

Kulkarni Onkar 1
Narreddy Suneetha 2
Zaveri Lamuk 1
Kalal Irawathy Goud 2
Tallapaka Karthik Bharadwaj 1
http://orcid.org/0000-0003-4340-6011 Sowpati Divya Tej 1
1 CSIR Centre for Cellular and Molecular Biology , Hyderabad India
2 Apollo Hospitals , Hyderabad, India
Address for correspondence: Divya Tej Sowpati, CSIR – Centre for Cellular and Molecular Biology, Uppal Road, Habsiguda, Hyderabad - 500007, India. Email – tej@ccmb.res.inThese authors contributed to the work equally

Electronic publication date: 2021 Feb 16
Electronic Location ID: ciab136
Received 2021 Jan 25
Copyright: © Infectious Diseases Society of America 2021.
Copyright year: 2021
License: This is an Open Access article distributed under the terms of the Creative Commons Attribution License (http://creativecommons.org/licenses/by/4.0/), which permits unrestricted reuse, distribution, and reproduction in any medium, provided the original work is properly cited.
License URL: https://creativecommons.org/licenses/by/4.0/

article-lifecycle: PAP
edited-state: accepted-manuscript

==============================
Evidence of SARS-CoV-2 reinfection without mutations in Spike protein

Onkar Kulkarni[1,#], Suneetha Narreddy[2,#], Lamuk Zaveri[1,#], Irawathy Goud Kalal[2], Karthik Bharadwaj Tallapaka[1], Divya Tej Sowpati[1]

1 - CSIR Centre for Cellular and Molecular Biology, Hyderabad India

2 - Apollo Hospitals, Hyderabad, India

### - These authors contributed to the work equally

*Address for correspondence:*

Divya Tej Sowpati, CSIR – Centre for Cellular and Molecular Biology, Uppal Road, Habsiguda, Hyderabad - 500007, India. Email – tej@ccmb.res.in

Accepted Manuscript

© Infectious Diseases Society of America 2021.
This is an Open Access article distributed under the terms of the Creative Commons Attribution License (http://creativecommons.org/licenses/by/4.0/), which permits unrestricted reuse, distribution, and reproduction in any medium, provided the original work is properly cited.

Dear Editor –

Several cases of SARS-CoV-2 reinfection have now been documented across the globe[1-3]. Recently, Selhorst et al[4] reported a case of reinfection despite the presence of neutralizing antibodies. Their study showed the presence of S477N, an immune escape mutation[5], in the Spike protein of the virus from the second episode. This conforms to the fact that most reported reinfections show the presence of at least one unique variation in structural proteins between episodes, particularly the Spike protein[6]. Here, we report two cases– one clear case and one possible case, of SARS-CoV-2 reinfection that were detected during routine surveillance. Of note, there was no difference in the Spike protein of the virus between episodes.

To establish the genetic diversity of the virus, the samples were sequenced on Oxford Nanopore MinION following the ARTIC v3 protocol[7], and further validated using Illumina sequencing. Genomes were assembled from raw data following a previously published method[8], covering most of the SARS-CoV-2 genome (Table S1). The details of the cases and subsequent analysis are outlined below:

Case 1:

A 61-year-old, apparently immunocompetent, male healthcare worker tested positive for SARS-CoV-2 using RT-PCR as part of contact tracing on 31st August 2020. After an episode of asymptomatic infection and home quarantine, he tested negative subsequently. With no travel history in between, he complained of weakness in 2nd week of November, and developed a cough 2 days later. He again tested positive for the virus on 14th November 2020. There were no other symptoms during this episode, and it was a mild disease overall. Sequencing revealed the presence of 10 unique variations between the viral genomes of both episodes (Fig 1, top, Table S2). No variation was observed in the Spike protein.

Case 2:

A 38-year-old male admitted to the hospital with symptoms of headache and fever tested positive for SARS-CoV-2 using RT-PCR on 4$^{th}$ November 2020. After a day of symptoms indicated above, the patient was symptom-free. On 22nd November 2020, the patient had fever again. Sample collected on that day tested positive for SARS-CoV-2. Other than 5 days of fever following the test, there were no symptoms during the second episode. There was a history of steroid usage for a diagnosis unrelated to COVID19 (Tuberculous meningitis). Subsequent analysis revealed the presence of 3 unique variations between both the episodes, and a large number of shared variants (Fig 1, bottom, Table S3). One of the unique variations in episode 2 was in the Spike protein; however, it was a synonymous change.

In summary, we report two cases of SARS-CoV-2 reinfections from India, along with corresponding whole genome sequencing data, confirmed using two orthogonal sequencing

Accepted Manuscript technologies. Nextstrain analysis[9] revealed that all the 4 viral genomes belonged to the 20B clade, and carry the D614G mutation in Spike. While case 1 is a clear case of reinfection backed by a negative test between episodes and 10 unique variations between the viral strains, case 2 remains a bit unclear; 3 unique variations in 18 days is higher than expected based on current estimates, but we cannot rule out the possibility of prolonged viral shedding and accelerated viral evolution due to immunocompromised state. However, in both cases, no variation from S or E genes was identified between reinfections. Taken together, our work provides evidence for a rare but distinct possibility of reinfection without changes in the Spike protein, and highlights the need for further research to understand the genetic and molecular underpinnings of COVID19 reinfections.

Accepted Manuscript

**Notes**

***Acknowledgments***: The authors thank Surabhi Srivastava for critical reading of the manuscript. Sofia Banu is acknowledged for the help in compiling variant information of previously reported reinfection cases.

***Financial support:*** This work was supported by the Council of Scientific and Industrial Research (CSIR) India (grant MLP0128).

***Potential conflicts of interest:*** SN reports personal speaker's fees from Reddy's Labs, outside the submitted work. All other authors report no potential conflicts of interest.

All authors have submitted the ICMJE Form for Disclosure of Potential Conflicts of Interest. Conflicts that the editors consider relevant to the content of the manuscript have been disclosed.

Accepted Manuscript

### References

1. Tillett, R.L., et al., *Genomic evidence for reinfection with SARS-CoV-2: a case study.* Lancet Infect Dis, 2021. **21**(1): p. 52-58.
2. Gupta, V., et al., *Asymptomatic reinfection in two healthcare workers from India with genetically distinct SARS-CoV-2.* Clin Infect Dis, 2020.
3. Lee, J.S., et al., *Evidence of Severe Acute Respiratory Syndrome Coronavirus 2 Reinfection After Recovery from Mild Coronavirus Disease 2019.* Clin Infect Dis, 2020.
4. Selhorst, P., et al., *Symptomatic SARS-CoV-2 reinfection of a health care worker in a Belgian nosocomial outbreak despite primary neutralizing antibody response.* Clin Infect Dis, 2020.
5. Liu, Z., et al., *Landscape analysis of escape variants identifies SARS-CoV-2 spike mutations that attenuate monoclonal and serum antibody neutralization.* bioRxiv, 2020.
6. Singh, P., et al. *Genome Evolution Analysis Resource for COVID19*. 2020; Available from: https://data.ccmb.res.in/gear19/reinfection.
7. Quick, J. *nCoV-2019 sequencing protocol v3 (LoCost) V.3*. 2020; Available from: https://www.protocols.io/view/ncov-2019-sequencing-protocol-v3-locost-bh42j8ye.
8. Banu, S., et al., *A Distinct Phylogenetic Cluster of Indian Severe Acute Respiratory Syndrome Coronavirus 2 Isolates.* Open Forum Infect Dis, 2020. **7**(11): p. ofaa434.
9. Hadfield, J., et al., *Nextstrain: real-time tracking of pathogen evolution.* Bioinformatics, 2018. **34**(23): p. 4121-4123.

Accepted Manuscript

**Figure legend:**

Figure 1: Whole genome sequence analysis of case 1 (top) and case 2 (bottom). Unique variants are represented by red bars whereas variants shared between both infection episodes are drawn as blue bars. Regions not covered well by sequencing are indicated in grey. An asterisk denotes clade defining variant.

Accepted Manuscript

Figure 1

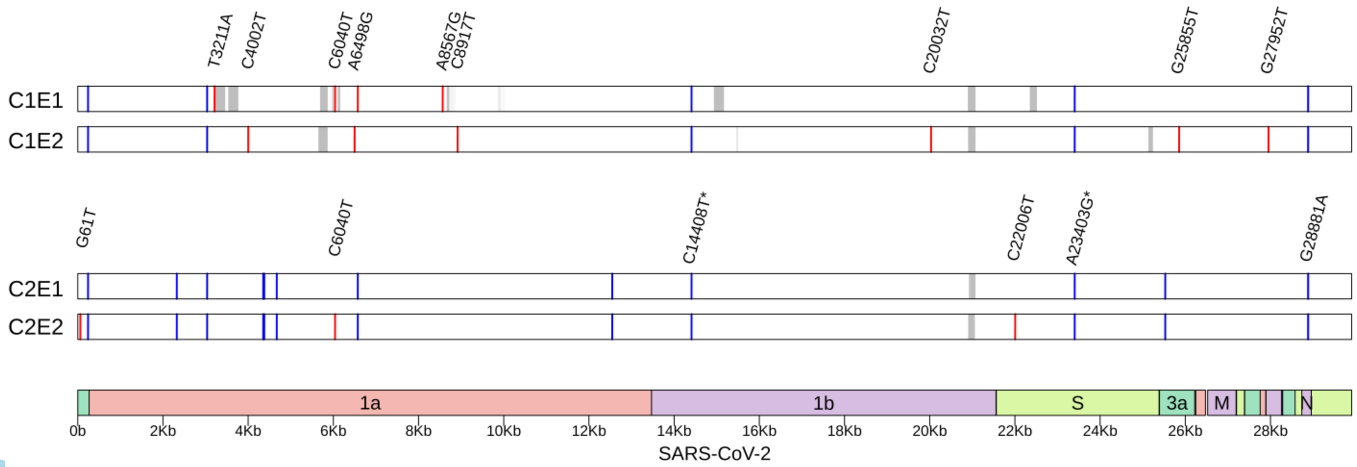

T3211A
C4002T
C6040T
A6498G
A8567G
C8917T
C20032T
G25855T
G27952T
C1E1
C1E2
G61T
C6040T
C14408T*
C22006T
A23403G*
G28881A
C2E1
C2E2
1a
1b
S
3a
M
N
0b
2Kb
4Kb
6Kb
8Kb
10Kb
12Kb
14Kb
16Kb
18Kb
20Kb
22Kb
24Kb
26Kb
28Kb
SARS-CoV-2
