# Evidence of Severe Acute Respiratory Syndrome Coronavirus 2 (SARS-CoV-2) Reinfection Without Mutations in the Spike Protein

**DOI:** 10.1093/cid/ciab136

**Published:** 2021-02-16

**Authors:** Onkar Kulkarni, Suneetha Narreddy, Lamuk Zaveri, Irawathy Goud Kalal, Karthik Bharadwaj Tallapaka, Divya Tej Sowpati

**Affiliations:** 1 Council of Scientific and Industrial Research (CSIR) Centre for Cellular and Molecular Biology, Hyderabad, India; 2 Apollo Hospitals, Hyderabad, India

To the Editor—Several cases of severe acute respiratory syndrome coronavirus 2 (SARS-CoV-2) reinfection have now been documented across the globe [1–3]. Recently, Selhorst et al [4] reported a case of reinfection despite the presence of neutralizing antibodies. Their study showed the presence of S477N, an immune escape mutation [5], in the spike protein (S) of the virus from the second episode. This conforms to the fact that most reported reinfections show the presence of at least 1 unique variation in structural proteins between episodes, particularly the spike protein [6]. Here, we report 2 cases—1 clear case and 1 possible case—of SARS-CoV-2 reinfection that were detected during routine surveillance. Of note, there was no difference in the spike protein of the virus between episodes.

To establish the genetic diversity of the virus, the samples were sequenced on Oxford Nanopore MinION (Oxford Nanopore Technologies, UK) following the ARTIC v3 protocol [7], and further validated using Illumina sequencing (Illumina Inc., USA). Genomes were assembled from raw data following a previously published method [8], covering most of the SARS-CoV-2 genome (Supplementary Table 1). The details of the cases and subsequent analysis are outlined below.

## CASE 1

**Figure 1.**
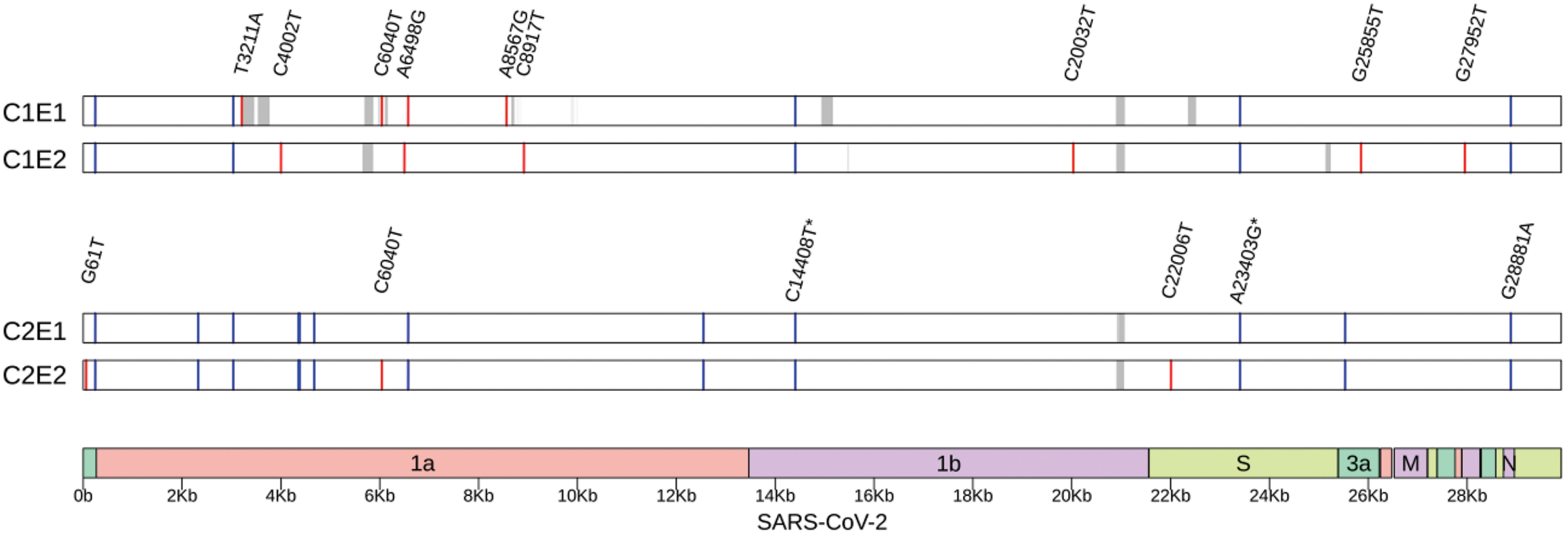
Whole-genome sequencing analysis of case 1 (top) and case 2 (bottom). Unique variants are represented by red bars, whereas variants shared between both infection episodes are drawn as blue bars. Regions not covered well by sequencing are indicated in gray. An asterisk denotes clade-defining variant. Abbreviation: SARS-CoV-2, severe acute respiratory syndrome coronavirus 2.

A 61-year-old, apparently immunocompetent, male healthcare worker tested positive for SARS-CoV-2 using reverse transcription–polymerase chain reaction (RT-PCR) as part of contact tracing on 31 August 2020. After an episode of asymptomatic infection and home quarantine, he tested negative subsequently. With no travel history in between, he complained of weakness in the second week of November and developed a cough 2 days later. He again tested positive for the virus on 14 November 2020. There were no other symptoms during this episode, and it was a mild disease overall. Sequencing revealed the presence of 10 unique variations between the viral genomes of both episodes (Figure 1, top; Supplementary Table 2). No variation was observed in the spike protein.

## CASE 2

A 38-year-old male admitted to the hospital with symptoms of headache and fever tested positive for SARS-CoV-2 using RT-PCR on 4 November 2020. After a day of symptoms indicated above, the patient was symptom free. On 22 November 2020, the patient again had fever. The sample collected on that day tested positive for SARS-CoV-2. Other than 5 days of fever following the test, there were no symptoms during the second episode. There was a history of steroid usage for a diagnosis unrelated to coronavirus disease 2019 (COVID-19) (tuberculous meningitis). Subsequent analysis revealed the presence of 3 unique variations between both of the episodes and a large number of shared variants (Figure 1, bottom; Supplementary Table 3). One of the unique variations in episode 2 was in the spike protein; however, it was a synonymous change.

In summary, we report 2 cases of SARS-CoV-2 reinfections from India, along with corresponding whole-genome sequencing data, confirmed using 2 orthogonal sequencing technologies. Nextstrain analysis [9] revealed that all of the 4 viral genomes belonged to the 20B clade, and carry the D614G mutation in spike. While case 1 is a clear case of reinfection backed by a negative test between episodes and 10 unique variations between the viral strains, case 2 remains a bit unclear; 3 unique variations in 18 days is higher than expected based on current estimates, but we cannot rule out the possibility of prolonged viral shedding and accelerated viral evolution due to immunocompromised state. However, in both cases, no variation from S or E genes was identified between reinfections. Taken together, our work provides evidence for a rare but distinct possibility of reinfection without changes in the spike protein, and highlights the need for further research to understand the genetic and molecular underpinnings of COVID-19 reinfections.

## Supplementary Data

Supplementary materials are available at *Clinical Infectious Diseases* online. Consisting of data provided by the authors to benefit the reader, the posted materials are not copyedited and are the sole responsibility of the authors, so questions or comments should be addressed to the corresponding author.

ciab136_suppl_Supplementary_Materials

## Notes

***Acknowledgments***. The authors thank Surabhi Srivastava for critical reading of the manuscript. Sofia Banu is acknowledged for the help in compiling variant information of previously reported reinfection cases.

***Financial support***. This work was supported by the Council of Scientific and Industrial Research (CSIR) India (grant number MLP0128).

***Potential conflicts of interest***. S. N. reports personal speaker’s fees from Reddy’s Labs, outside the submitted work. All other authors report no potential conflicts. All authors have submitted the ICMJE Form for Disclosure of Potential Conflicts of Interest. Conflicts that the editors consider relevant to the content of the manuscript have been disclosed.
