# Supplementary material for: Evidence of Severe Acute Respiratory Syndrome Coronavirus 2 (SARS-CoV-2) Reinfection Without Mutations in the Spike Protein
Source: Clin Infect Dis. 2021 Feb 16;73(5):e1239–41. doi: 10.1093/cid/ciab136 (PMC7929054; doi:10.1093/cid/ciab136)
Supplement: ciab136_suppl_Supplementary_Materials [file ciab136_suppl_supplementary_materials.docx]

Table S1: Genome coverage and other details of the reinfection samples

| **Samples** | **Average Coverage** | **% Genome Covered** | **GISAID Accession ID** | **GISAID Submission Date** |
| --- | --- | --- | --- | --- |
| C1E1 | 1380 | 95.14 | EPI_ISL_909955 | 2021-02-01 |
| C1E2 | 5874 | 98.28 | EPI_ISL_909956 | 2021-02-01 |
| C2E1 | 8427 | 99.49 | EPI_ISL_909957 | 2021-02-01 |
| C2E2 | 8618 | 99.47 | EPI_ISL_909958 | 2021-02-01 |

Table S2: Variant list of both episodes of case 1. Unique variants between episodes are highlighted in yellow.

| **Position** | **Ref** | **Episode1** | **Episode2** |
| --- | --- | --- | --- |
| 241 | C | T | T |
| 3037 | C | T | T |
| 3211 | T | A | T |
| 4002 | C | C | T |
| 6040 | C | T | C |
| 6498 | A | A | G |
| 6573 | C | T | C |
| 8567 | A | G | A |
| 8917 | C | C | T |
| 14408 | C | T | T |
| 20032 | C | C | T |
| 23403 | A | G | G |
| 25855 | G | G | T |
| 27952 | G | G | T |
| 28881 | G | A | A |
| 28882 | G | A | A |
| 28883 | G | C | C |

Table S3: Variant list of both episodes of case 2. Unique variants between episodes are highlighted in yellow.

| **Position** | **Ref** | **Episode1** | **Episode2** |
| --- | --- | --- | --- |
| 61 | G | G | T |
| 241 | C | T | T |
| 2325 | C | T | T |
| 3037 | C | T | T |
| 4354 | G | A | A |
| 4378 | T | C | C |
| 4672 | C | T | T |
| 6040 | C | C | T |
| 6573 | C | T | T |
| 12547 | A | T | T |
| 14408 | C | T | T |
| 22006 | C | C | T |
| 23403 | A | G | G |
| 25528 | C | T | T |
| 28881 | G | A | A |
| 28882 | G | A | A |
| 28883 | G | C | C |
